# Fecal microbiota changes associated with pathogenic and non-pathogenic diarrheas in foals

**DOI:** 10.1186/s13104-025-07110-9

**Published:** 2025-01-23

**Authors:** Yijun Shi, Elizabeth A. Maga, Michael J. Mienaltowski

**Affiliations:** Department of Animal Science, University of California Davis, 2251 Meyer Hall, One Shields Ave, Davis, CA 95616 USA

**Keywords:** Equine, Foal, Diarrhea, Clostridium, Microbiota

## Abstract

**Objectives:**

Diarrhea is a common disease that could threaten the welfare of newborn foals. While there are several forms of foal diarrhea, the etiologies can be considered known pathogenic or non-pathogenic in nature. Moreover, there are likely differences in the composition of microbial populations in the gastrointestinal tracts of foals depending upon the etiology of diarrhea. Our study aims to examine the microbial population in the feces of foals with both pathogenic and non-pathogenic diarrheas to discern differences in their microbial compositions.

**Results:**

Foal diarrhea samples tested positive or negative for common equine neonatal diarrhea pathogens by diagnostic polymerase chain reaction (PCR), which allowed for samples to be segregated as pathogenic or non-pathogenic. Pathogenic samples tested positive for combinations of *Clostridium perfringens* and/or *Clostridioides difficile* toxins. As a result, significantly higher alpha diversity was seen in the non-pathogenic samples than in pathogenic ones. Sequencing of the V4 domains of bacterial 16 S rRNA genes demonstrated that non-pathogenic samples had more alpha diversity. Furthermore, eight microbial families and eleven genera showed significant differences in their abundances between pathogenic and non-pathogenic diarrhea samples.

**Supplementary Information:**

The online version contains supplementary material available at 10.1186/s13104-025-07110-9.

## Introduction

Diarrhea is prevalent in foals, occurring in roughly 50% of neonates from birth to weaning [1, 2]. Immediate evaluation, veterinary medical intervention, and management are generally prescribed because complications associated with diarrhea lead to significant threats to a foal’s welfare [1, 2]. There can be great variation in the microbial populations found in the gastrointestinal tracts of horses, including foals, in relation to the etiologies of diarrhea and associated dysbiosis [2–7]. In this study, we examined the differences between fecal microbial populations in foals afflicted with pathogenic and non-pathogenic diarrheas. We hypothesized that microbial populations would differ in fecal samples from foals with pathogenic diarrheas and non-pathogenic diarrheas. To examine our hypothesis, fecal samples were collected from foals with diarrhea. The samples were sent to a diagnostic laboratory for the detection of common equine diarrhea pathogens. Additionally, microbial DNA was isolated from the samples for PCR amplification of the V4 domain of bacterial 16 S rRNA genes; amplicons then were subjected to next-generation sequencing. Subsequently, bacterial compositions from each sample were analyzed to elucidate which microbial populations were associated with pathogenic and non-pathogenic diarrheas.

## Materials and methods

### Fecal sample collection and diagnostic qPCR

Research was conducted with the approval of the UC Davis Institutional Animal Care and Use Committee. Voluntarily-voided fecal diarrhea samples, characterized as loose and watery stools, were collected at three farms from 19 Quarterhorse and Thoroughbred foals aged 3-143 days (Table S1). Then the fecal samples were swabbed with flocked swabs (FLOQSwabs 552 C, Copan Diagnostics Inc.). Swabs and samples were stored at -80℃. Swabs were submitted to the Real-time PCR Research and Diagnostics Core Facility at the UC Davis School of Veterinary Medicine for VMTH Extended Foal Neonate Panel real-time PCR testing, which screens for Equine Coronavirus, Equine Rotavirus, *Clostridioides difficile* toxin A and B, *Lawsonia intracellularis*, *Salmonella* spp, *Cryptosporidium* spp, *Rhodococcus equi*, *Clostridium perfringens* alpha toxin, *Clostridium perfringens* beta toxin, *Clostridium perfringens* beta2 toxin, *Clostridium perfringens* cytotoxin netF, *Clostridium perfringens* enterotoxin cpe, and *Neorickettsia risticii*. Diarrhea samples were then segregated as positive or negative for pathogenic species based on panel results.

### Microbial DNA isolation and DNA sequencing

DNA was isolated from fecal samples using the Quick-DNA Fecal/Soil Microbe Miniprep Kit (Zymo Research, #D6010) [3, 8]. DNA concentrations were determined using a NanoDrop UV spectrophotometer (ThermoFisher Scientific). The V4 domain of bacterial 16 S rRNA genes and a unique 8 bp barcode on each forward primer for sample identification were amplified with PCR in triplicate using GoTaq 2X Green Master Mix (Promega) as previously described [3, 9]. Reactions were: (1) an initial step at 94 °C for 3 min; (2) then 35 cycles at 94 °C for 45 s, 50 °C for 1 min, and 72 °C for 90 s; and (3) a final extension at 72 °C for 10 min. Combined triplicates for each sample were screened for PCR amplification success via agarose gel electrophoresis; no amplification was detected in negative (no DNA) controls. PCR products were combined in equal volumes and purified using QIAGEN’s PCR Purification Kit. Combined barcoded libraries were submitted to the UC Davis Genome Center DNA Technologies Core for 250 bp paired-end sequencing using the Illumina MiSeq platform. Raw sequence data are freely available at the Sequence Read Archive (SRA): Bio Project PRJNA1079832.

### Sequencing analysis

Sequencing files were demultiplexed using *Barcode Splitter* in Galaxy [10, 11]. Demultiplexed FASTQ files were imported into *BioConductor* v3.12 and *DADA2* v1.18 in *RStudio* v1.4 using *R* v4.1.0; reads were applied to *DADA2* and *phyloseq* package v1.38 with *Biostrings* to generate an amplicon sequence variant (ASV) table [8, 12–17]. These packages were used to produce data tables with taxa annotations and discern alpha diversity. Further analyses were performed using the Marker Data Profiling tool of the online *MicrobiomeAnalyst* program; raw abundance genera-level data tables with metadata were submitted, and filtering parameters were applied (minimum count: 4; low count filter: prevalence 20%; low variance filter: 10%, inter-quartile range; data normalization with rarefaction and total sum scaling) [18–20]. *MicrobiomeAnalyst* was used to display beta diversity as Principal Coordinate Analysis using the Bray-Curtis Index at the genus level, as well as to perform heat tree analysis and for determinations of linear discriminant analysis effect size (LEfSe) [21, 22].

### Statistical analysis

Species richness of pathogenic and non-pathogenic diarrheas were examined with four different alpha diversity indices: Chao 1, Shannon, Simpson, and Fisher. Graphs were generated using GraphPad Prism version 10.1.2 for Windows (GraphPad Software, USA). Further statistical analysis on microbial abundance was conducted individually at the phylum, family, and genus taxonomic levels using ASV counts from sequencing data. Data were organized using Microsoft Excel version 2016 and subjected to statistical testing for the non-parametric Mann-Whitney test, using GraphPad Prism with a significance threshold set at *p* ≤ 0.05. Pearson’s r correlations of day and alpha diversity indices for pathogenic and non-pathogenic diarrheas were determined using *jamovi* [23].

## Results

**Fig. 1:**
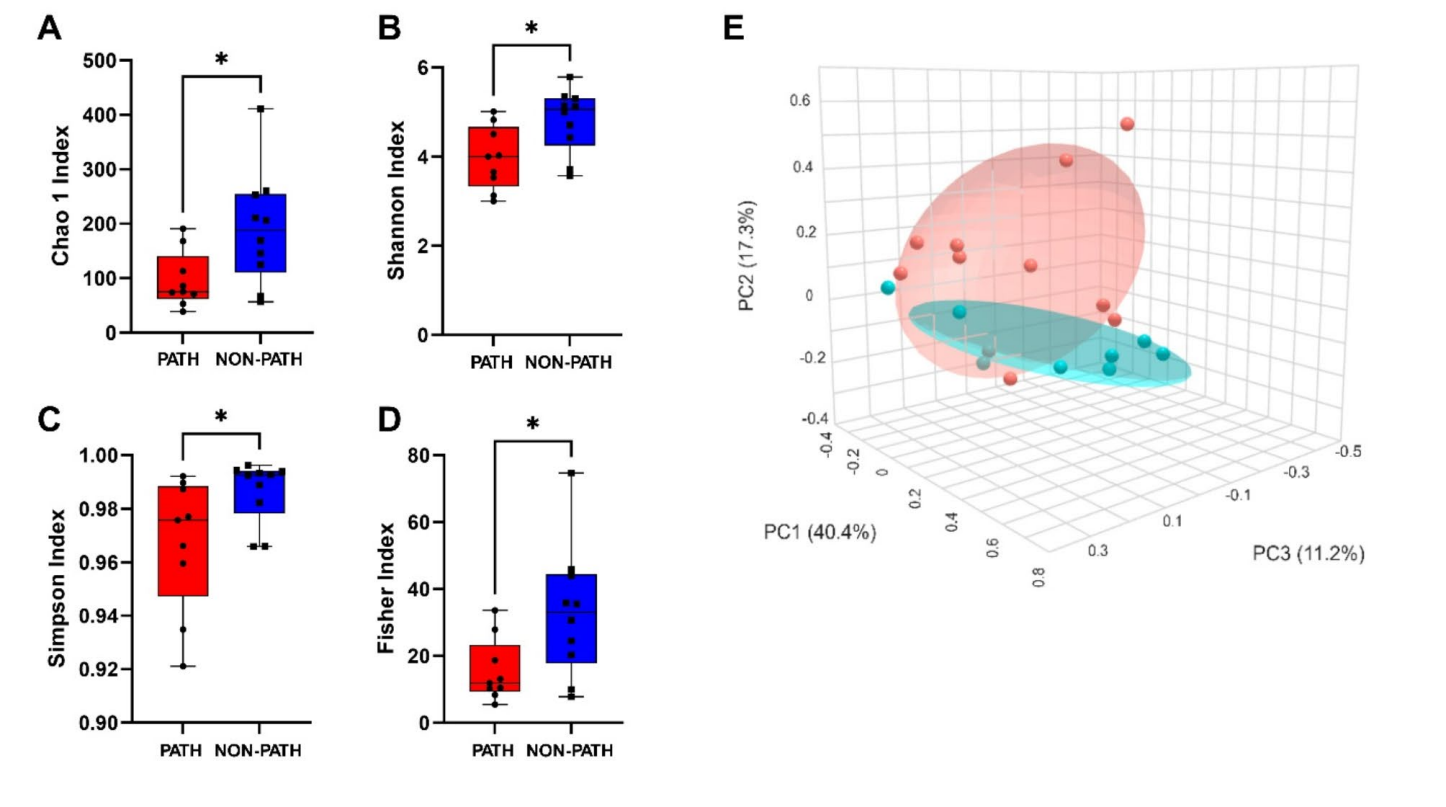
Diversity analyses of pathogenic and non-pathogenic diarrhea samples. Alpha diversity was analyzed using **(A)** Chao 1, **(B)** Shannon, **(C)** Simpson, and **(D)** Fisher indices. The red box plots represent diarrhea samples with positive diagnostic detection of pathogenic microbes, and the blue box plots represent samples with no pathogens detected by qPCR. *n* = 9–10 samples per group; statistical analyses performed using Mann-Whitney test with * representing significance (*p* ≤ 0.05). **(E)** Beta diversity was examined via principal coordinate analysis of microbial genera using the Bray-Curtis Index for the distance method with samples divided by detectable pathogen status as well as PERMANOVA statistics. Red points represent samples with positive diagnostic detection of pathogenic microbes; blue points represent samples with no pathogens detected

From the qPCR diagnostic results, 10 fecal samples were reported negative for pathogens and 9 samples received positive amplification for combinations of *Clostridium perfringens* and/or *Clostridioides difficile* toxins; other pathogens from the Extended Foal Neonate Panel were not detected (Table S1). The mean ASVs mapped across taxonomy hierarchy down to specific genera were 8,062 ± 2,266 (range: 4,375 − 11,295). Because of unequal sequencing depth between samples, data were rarefied. In the assessment of alpha diversity of species richness, all four alpha diversity indices demonstrated that non-pathogenic samples had significantly more microbial diversity than pathogenic samples (Fig. 1A, B, C and D). Furthermore, diarrhea diminished age-related correlations with increased diversity, particularly for pathogenic samples (Table S2). A principal coordinate analysis indicated that non-pathogenic samples were more closely clustered together while samples from pathogenic diarrheas were more spread across the top three coordinates (Fig. 1E).

The relative abundance of 18 phyla, 83 families, and 184 genera were examined. Seven out of 18 phyla represented the majority of the microbes (Figure S1). *Firmicutes* was the most abundant phylum, accounting for 47.47% of the pathogenic microbial population and 54.98% of the non-pathogenic microbial population. *Bacteroidota* and *Proteobacteria* followed with *Bacteroidota* comprising 21.9% of the pathogenic population and 20.96% of the non-pathogenic population and *Proteobacteria* comprising 22.48% of the pathogenic population and 13.34% of the non-pathogenic population. For the other four phyla that comprised the majority of the microbes detected across all samples (*Actinobacteriota*, *Desulfobacterota*, *Fusobacteriota*, and *Verrucomicrobiota*), their abundances ranged from 0.23 to 4.75% of the bacteria present. No significant differences were seen between groups at the phylum level.

**Fig. 2:**
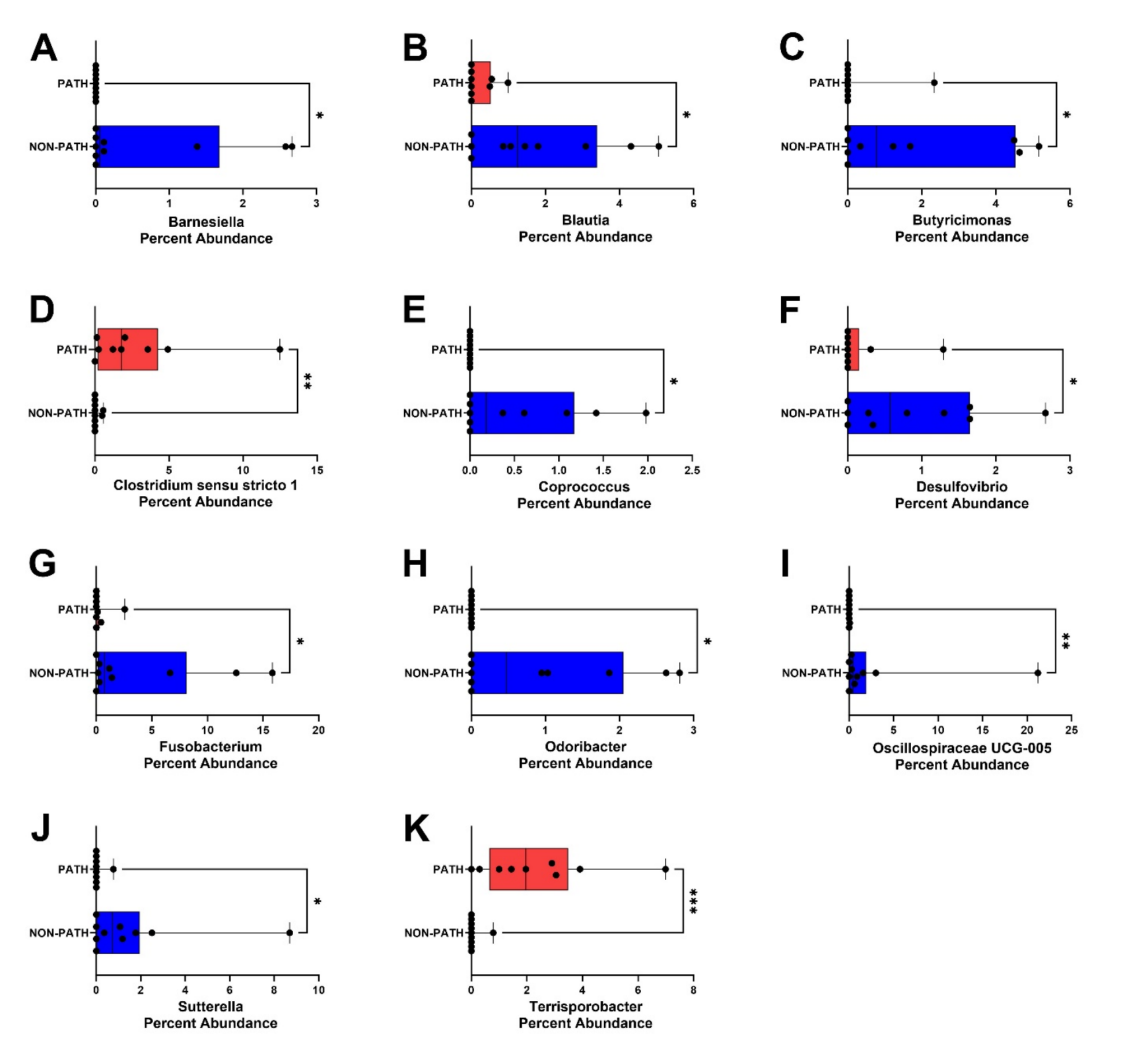
Distributions of microbial genera. Differences in abundance were seen across genera: **(A)***Barnesiella*, **(B)***Blautia*, **(C)***Butyricimonas*, **(D)***Clostridium sensu stricto 1*, **(E)***Coprococcus*, **(F)***Desulfovibrio*, **(G)***Fusobacterium*, **(H)***Odoribacter*, **(I)***Oscillospiraceae UCG-005*, **(J)***Sutterella*, **(K)***Terrisporobacter.* Data displayed in box and whisker plot with *n* = 9–10 samples per group; statistical analyses performed using Mann-Whitney test with significance represented as * (*p* ≤ 0.05), ** (*p* ≤ 0.01), and *** (*p* ≤ 0.001)

**Fig. 3:**
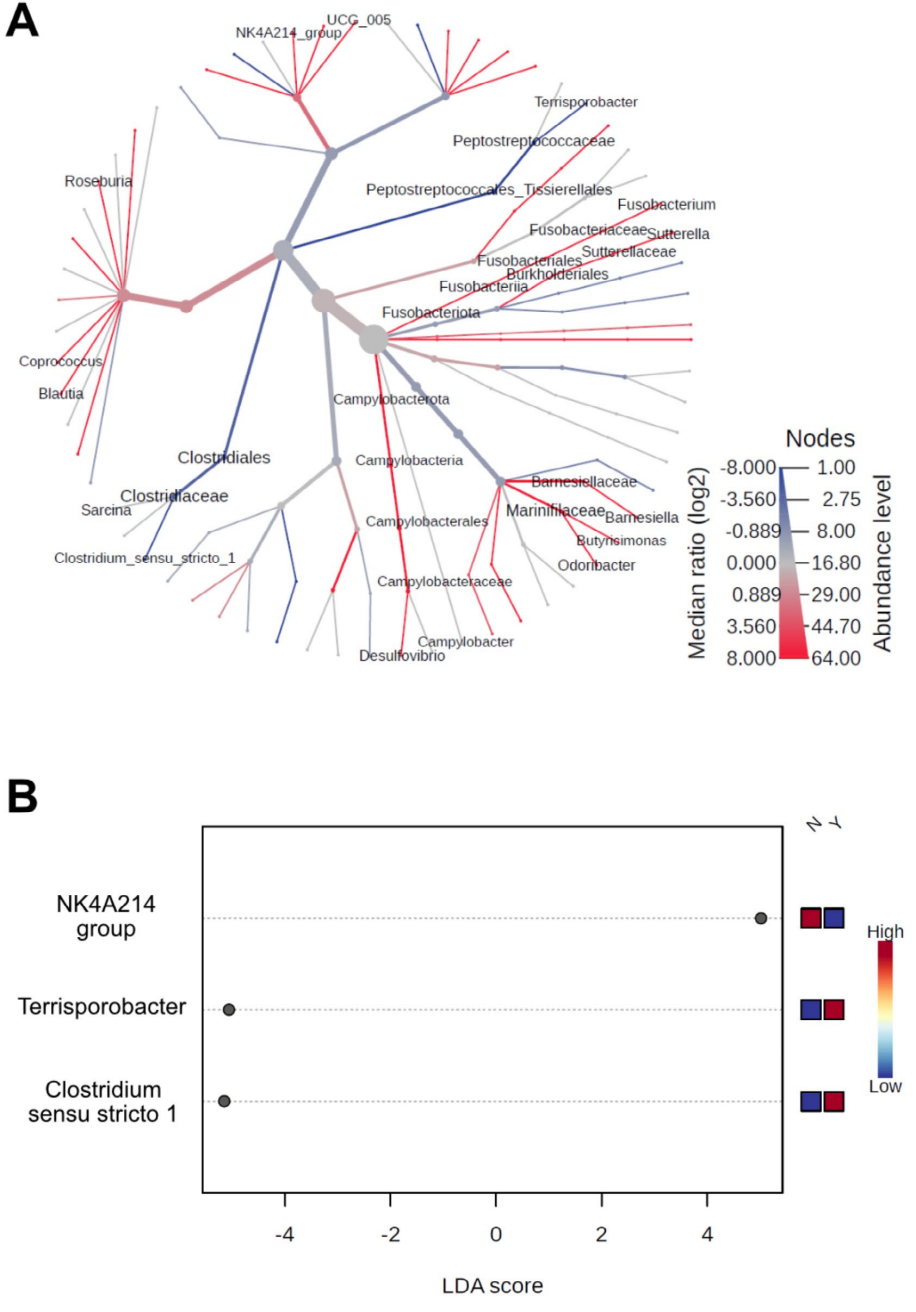
Heat tree and LEfSe analyses highlight distinctions in microbial populations between groups. MicrobiomeAnalyst was used to perform **(A)** heat tree and **(B)** linear discriminant analysis (LDA) effect size (LEfSe) analyses. Heat tree analysis detected differences in 15 genera, using a Wilcox rank-sum test followed by a Benjamini-Hochberg (FDR) correction for multiple testing; red lines represent microbes with greater abundance in non-pathogenic diarrheas and blue lines represent microbes with greater abundance in pathogenic diarrheas. LEfSe analysis found three genera with distinctly different abundances. Both analyses are based upon an FDR-adjusted p-value cutoff of *p* ≤ 0.10

We found that eight families and eleven genera demonstrated differences in relative abundance between pathogenic and non-pathogenic groups (Fig. 2, S2). Families *Barnesiellaceae*,* Christensenellaceae*,* Marinifilaceae*,* Succinivibrionaceae*,* and Sutterellaceae* exhibited greater abundance within non-pathogenic fecal samples (Figure S2A, S2D, S2E, S2G, S2H). The three remaining families *Clostridiaceae*,* Enterobacteriaceae*, and *Micrococcaceae* were present in higher abundance in pathogenic samples (Figure S2B, S2C, S2F). Genera *Barnesiella*,* Blautia*,* Butyricimonas*,* Coprococcus*,* Desulfovibrio*,* Fusobacterium*,* Odoribacter*,* Oscillospiraceae UCG-005*, and *Sutterella* all exhibited greater abundance in non-pathogenic diarrhea fecal samples (Fig. 2A-C and E-J). Genera *Clostridium sensu stricto 1* and *Terrisporobacter* exhibited higher abundance in pathogenic samples (Fig. 2D and K). Differences in microbial abundances for many of the above-mentioned microbes were confirmed by heat tree analysis within *MicrobiomeAnalyst* (Fig. 3A). LEfSe analysis revealed that *Ruminococcaceae* NK4A214 group microbes were found in greater abundance in non-pathogenic diarrhea samples while pathogenic diarrheas had a greater abundance of microbes in the genera *Clostridium sensu stricto 1* and *Terrisoportobacter* (Fig. 3B).

## Discussion

We hypothesized that foals with pathogenic diarrheas would exhibit distinct compositions of microbial populations compared to those experiencing non-pathogenic diarrheas. Based upon the fecal microbial populations examined, our sequencing data supported the hypothesis, particularly when examining alpha diversity and in comparing microbial populations at the family and genus levels.

Fecal samples from non-pathogenic diarrheas had greater alpha diversity than those of pathogenic diarrheas. However, an analysis of beta diversity did not reveal distinct clustering between the two sample categories, indicating that while differences were seen between samples, microbial populations had a high degree of resemblance across samples. Previously we demonstrated that microbial diversity increases with aging in foals [3, 8]. Others have shown that greater alpha diversity is seen in fecal samples and rectal swabs of healthy adult horses relative to diarrheic adult horses [4, 5]. In this study, we found that pathogenic diagnoses were associated with dysbiosis as could be implied with decreased microbial diversity.

For the feces sampled from the diarrheic foals, differences in microbial populations were first seen at the family level. Eight families exhibited significant differences in abundance between pathogenic and non-pathogenic diarrheas with five families observed to have a higher abundance in non-pathogenic diarrheas and three families having greater abundance in pathogenic diarrheas. Furthermore, there were eleven genera with significant differences between groups of diarrheic foals - nine genera exhibited higher abundance in non-pathogenic cases while two genera showed higher abundance for pathogenic diarrheas. From the differences in microbial abundance seen at the family level, two microbial families seemed to define the pathogenic samples. The family with the greatest abundance in pathogenic samples was *Enterobacteriaceae*, which has been associated with equine colitis specifically and dysbiosis in general [3, 24, 25]. In livestock, including foals, many species from the family *Enterobacteriaceae* are often found to have some abundance in bacterial diarrheas, though a causative etiology has not been established [6]. Furthermore, diarrheas in foals are commonly caused by microbes from the family *Clostridiaceae* [1, 26]; all of the foals considered to have pathogenic diarrhea tested positive by qPCR for strains of *Clostridium perfringens* and/or *Clostridioides difficile* in this study.

We also examined the impact of differences in abundance in genera between pathogenic and non-pathogenic samples. Genus *Barnesiella*, for example, has the function of restricting the growth of pathogens which is likely relevant to its greater abundance in foals with non-pathogenic diarrhea [27]. Furthermore, *Blautia*, *Butyricimonas*, *Coprococcus*, *Desulfovibrio*, and *Sutterella* are considered to have positive effects on the health of the host’s gastrointestinal or immune systems, so their lower abundance in foals with pathogenic diarrhea could be related to their role in gastrointestinal health [5, 7, 28–29]. *Odoribacter* abundance has been correlated with age in equids and is a prevalent genus in young donkeys aged from 1 to 3 months [30]. Similarly, *Oscillospiraceae UCG-005* abundance also has a positive correlation to days post-foaling [8]. Since our target foals aged from 3 to 143 days, the abundance of *Odoribacter* and *Oscillospiraceae UCG-005* in the non-pathogenic population could be related to foal age. There is a species of *Odoribacter* (*splanchnicus*) that is capable of limiting colitis in a mouse model, which might explain the genus’s association in non-pathogenic fecal samples; however, our study did not have the resolution to specifically quantify such species [31, 32]. Moreover, a previous study on horses with diarrhea recorded an increase in *Clostridium sensu stricto* which was suspected to be associated with diarrhea and a less healthy gastrointestinal condition, and could account for *Clostridium sensu stricto 1*’s high abundance in pathogenic diarrhea samples [33]. *Terrisporobacter* was the other genus for which there was a greater abundance of microbes in the pathogenic samples; this genus is so closely related to *Clostridium* that species of *Clostridium* have been reclassified as *Terrisporobacter* [34].

In this study, we noted differences in microbial populations between diarrhea samples of foals who tested positive by qPCR for pathogenic microbial strains *Clostridioides difficile* and *Clostridium perfringens*, relative to those diarrhea samples testing negative for a panel of equine diarrhea pathogens. We also found that microbial diversity was impacted in those samples testing positive for a pathogen. Therefore, while diarrhea is often associated with dysbiosis and changes in gut microbiota, it is important to consider that pathogens could more significantly impact GI disturbances.

### Limitations

The study had several limitations. The small sample set revealed positive diagnostic findings for some samples for two primary pathogens *Clostridioides difficile* and *Clostridium perfringens*. Other pathogenic etiologies could have been possible but were not tested within the diagnostic qPCR panel. Furthermore, some pathogens are shed intermittently, like *Salmonella* species; thus, while not detected in the samples studied, their presence cannot truly be ruled out without repeat testing, which was not done in this study. Likewise, “no diarrhea” or healthy control samples were not collected from the horses to confirm presence or absence of microbial populations in other states of health. Finally, determining the cause of pathogenic diarrheas via next-generation sequencing technology likely requires better resolution of sequencing or microbial transcript sequencing and thus better detection of pathogens in the diarrhea cases.

## Electronic supplementary material

Below is the link to the electronic supplementary material.

Supplementary Material 1

## Data Availability

The datasets for this study are available in the NCBI Short Read Archive repository under accession BioProject PRJNA1079832.

## Abbreviations

ASV: Amplification sequence variant
DNA: Deoxyribonucleic Acid
FDR: False discovery rate
GI: Gastrointestinal
PCA: Principal component analysis
qPCR: Quantitative polymerase chain reaction
rRNA: Ribosomal RNA
