# Supplementary material for: Fecal microbiota changes associated with pathogenic and non-pathogenic diarrheas in foals
Source: BMC Res Notes. 2025 Jan 23;18:34. doi: 10.1186/s13104-025-07110-9 (PMC11760091; doi:10.1186/s13104-025-07110-9)
Supplement: Supplementary file 1 — Supplementary Material 1 [file 13104_2025_7110_MOESM1_ESM.pdf]

Supplementary Materials

Table S1. Diagnostic Quantitative PCR Results for Diarrhea Samples.

Foal ID	Farm	DOB	Sample Day	qPCR Negative	Clostridium perfringens alpha toxin +	Clostridium perfringens beta2 toxin +	Clostridium perfringens enterotxin cpe	Clostridiodes difficile toxin A and B+
11	1	6/1/2016	9	X				
12	2	3/12/2016	7				X	
14	2	3/12/2016	7	X				
15	2	3/21/2016	7		X			X
19	2	3/18/2016	3		X			
20	2	3/19/2016	3		X			
24	2	4/4/2016	7		X			X
25	2	4/6/2016	7			X		X
32	2	4/9/2016	3		X			X
35	2	4/21/2016	7	X				
36	2	4/24/2016	28	X				
37	2	4/25/2016	3					X
38	2	5/6/2016	7	X				
999	3	4/18/2016	143	X				
106	1	5/11/2017	8		X			X
110	2	3/31/2017	12	X				
113	2	3/31/2017	12	X				
115	2	4/3/2017	15	X				
116	2	4/5/2017	13	X				

Table S2. Diversity-Day Pearson's Correlations.

	Pathogenic Samples		Non-pathogenic Samples	
	**Pearson's r**	**p-value**	**Pearson's r**	**p-value**
**Chao**	0.338	0.374	**0.771**	**0.009**
**Shannon**	0.152	0.696	0.534	0.112
**Simpson**	-0.107	0.784	0.357	0.311
**Fisher**	0.361	0.341	**0.782**	**0.008**

Pearson's correlations were applied to the nine pathogenic samples and eleven pathogenic samples, comparing alpha diversity indices to days of age of foal at time of diarrhea sample collection. Significant Pearson's correlations are bolded ($p \leq 0.05$)..

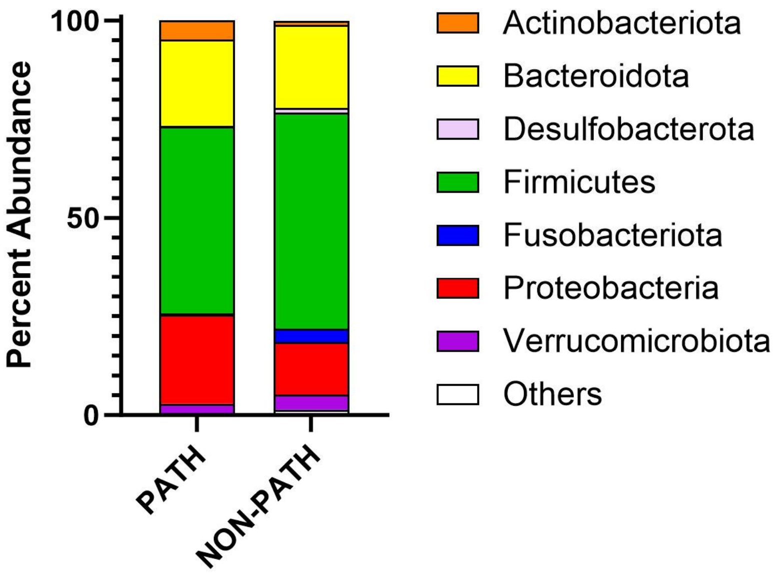

Figure S1. Stacked bar graph of most abundant microbial phyla found in samples. The seven most abundant microbial phyla detected in the amplicon sequencing are depicted. In comparing between groups, no phyla demonstrated significant differences in abundance. n=9-10 samples per group; statistical analyses performed using the Mann-Whitney test.

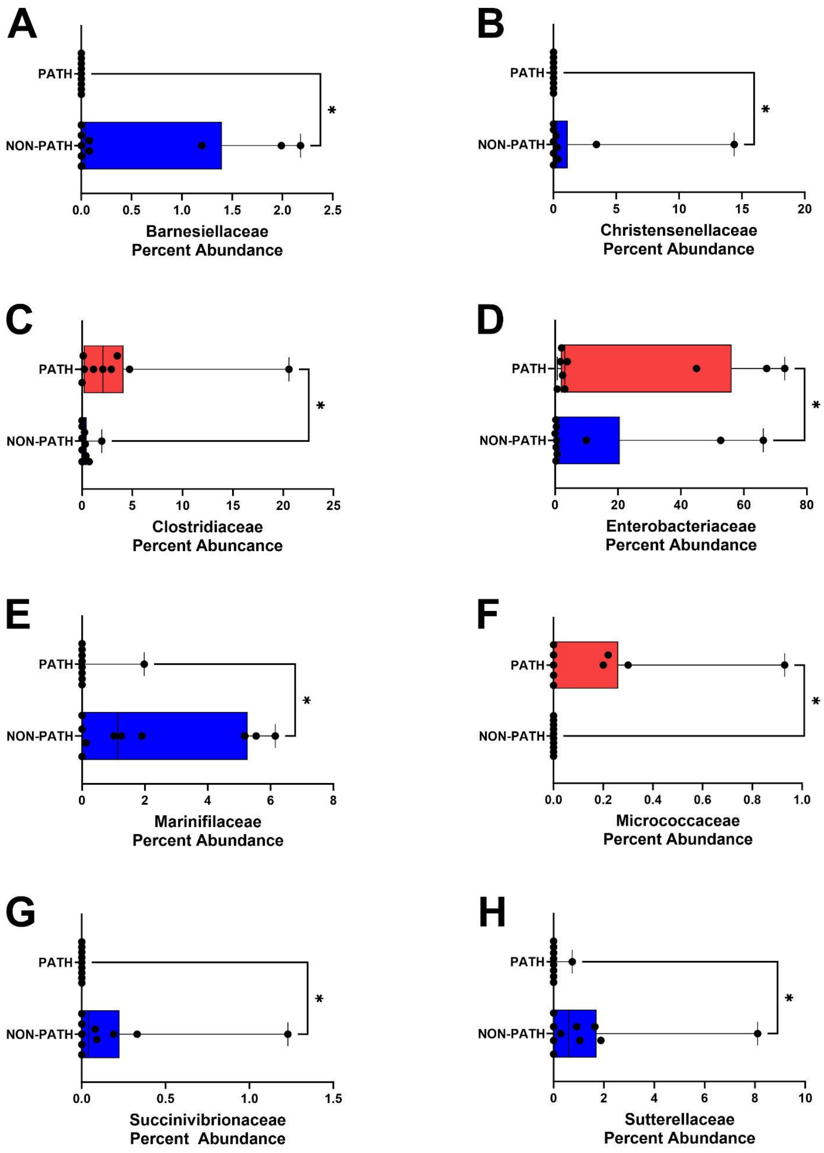

Figure S2. Distributions of microbial families. Differences in abundance were seen across families: **(A)** *Barnesiellaceae*, **(B)** *Christensenellaceae*, **(C)** *Clostridiaceae*, **(D)** *Enterobacteriaceae*, **(E)** *Marinifilaceae*, **(F)** *Micrococcaceae*, **(G)** *Succinivibrionaceae*, and **(H)** *Sutterellaceae*. Data displayed in box and whisker plot with n=9-10 samples per group; statistical analyses performed using Mann-Whitney test with significance represented as * ($p \leq 0.05$).
